# Decrease in reaction time for volleyball athletes during saccadic eye movement task: A preliminary study with evoked potentials

**DOI:** 10.1371/journal.pone.0290142

**Published:** 2024-07-03

**Authors:** Élida Costa, Mariana Gongora, Juliana Bittencourt, Victor Marinho, Mauricio Cagy, Silmar Teixeira, Eduardo Nicoliche, Isabelle Fernandes, Caroline Machado, Jacob Wienecke, Pedro Ribeiro, Daya S. Gupta, Bruna Velasques, Henning Budde

**Affiliations:** 1 Laboratory of Neurophysiology and Neuropsychology of Attention, Institute of Psychiatry of Federal University of Rio de Janeiro, Rio de Janeiro, Brazil; 2 School of Physical Education and Sport, Federal University of Rio de Janeiro, Rio de Janeiro, Brazil; 3 Brain Mapping and Sensory Motor Integration Laboratory, Institute of Psychiatry of Federal University of Rio de Janeiro, Rio de Janeiro, Brazil; 4 Veiga de Almeida University, Rio de Janeiro, Brazil; 5 Neuro-innovation Technology & Brain Mapping Laboratory, Federal University of Delta do Parnaíba, Parnaíba, Brazil; 6 Biomedical Engineering Program, Federal University of Rio de Janeiro, Rio de Janeiro, Brazil; 7 Department of Nutrition, Exercise and Sports, University of Copenhagen, Copenhagen, Denmark; 8 School of Pharmacy, South University, Savannah, Georgia, United States of America; 9 Faculty of Human Sciences, Institute for Systems Medicine, MSH Medical School Hamburg, Hamburg, Germany; La Sapienza University of Rome, ITALY

## Abstract

**Aim:**

This preliminary study investigated the differences in event-related potential and reaction time under two groups (athletes *vs*. non-athletes).

**Material and methods:**

The P300 was analyzed for Fz, Cz, and Pz electrodes in thirty-one healthy volunteers divided into two groups (volleyball athletes and non-athletes). In addition, the participants performed a saccadic eye movement task to measure reaction time.

**Results:**

The EEG analysis showed that the athletes, in comparison to the no-athletes, have differences in the P300 in the frontal area (p = 0.021). In relation to reaction time, the results show lower reaction time for athletes (p = 0.001).

**Conclusions:**

The volleyball athletes may present a greater allocation of attention during the execution of the inhibition task, since they have a lower reaction time for responses when compared to non-athletes.

## 1. Introduction

Skilled performance plays a key role in improving cognitive aspects such as attention, memory, and decision-making [1,2]. In this context, systematic sports practices such as tennis, baseball, or volleyball involve changes in neural architecture, connectivity in response to environmental stimulation, or extensive practice [3]. Some neuroimaging and neurophysiological studies in athletes have shown that neural processing during cognition and decision-making for motor acts are modulated by long-term perceptual-motor training and may present patterns of neural inputs and outputs consistent with neural efficiency [4–7]. Although it is evident those sports are related to physical and mental benefits, it is necessary to understand how regular sports practice interferes with cognitive processes [8,9]. Thus, it can provide a more comprehensive understanding of the differences in relation to neural processing for attentional level, with a greater focus on high-performance athletes when compared to non-athletes and beginning athletes [4].

The EEG analysis by event-related potential (ERP) P300 simultaneously with cognitive tasks is a method to evaluate patterns of cognitive processing, components elicited in the process of decision-making [10–12]. The concomitant assessment enhances the functional diagnosis of the central visual pathway, as well as a tool for studying the endogenous potential in the cortical mechanisms of visual perceptual processing, since your occurrence links not to the physical attributes of a stimulus but to a person’s reaction to it [13]. In particular, the P300 supports the evidence about the level and orientation of attention, contextual updating, modulation, and response resolution by latency and amplitude measures [14,15]. The P300 amplitude is directly proportional to the subject’s attention, and the increase in the P300 latency may indicate prolonged temporal processing during complex cognitive information [16–18].

Athletes’ cognitive engagement is necessary for high performance in several sports, in focus the volleyball, has been studied extensively [19,20]. The practice of this sport requires a high attention process due to vast exposure to dynamic visual stimuli. Therefore, the ability to have efficient attention is an essential factor for the success of these elite athletes [21]. In the sense, the premotor theory of attention defines the attention processes in response to visual-motor stimuli, which states shifts of attention occur by planning goal-directed actions such as eye movements and reaches. The spatial attention and motor preparation could be structurally and functionally equivalents, and may share neural networks when the situation involves directly planning an action mainly to the oculomotor system [4,14,15,22,23].

Previous studies demonstrated that P300 ERPs components in volleyball athletes were significantly improve when to compare with non-athletes [21,23]. These findings evidenced that participating in reactive, fast-paced sports has a greater effect on visual processing effectiveness than participating in other kinds of sports [3]. The understanding of this athletes performance during the sport can be supported on the premotor theory assumes that goal-driven by attention level is a dynamic mechanism, where an ocular motor system formed can brings the target into the fovea [24]. The substantial difference between driven "movement" and eye movement can be efficiently measured through saccadic eye movement (SEM) paradigm, because it allows investigating the first stages of visual processing and its relationship with attention [25–27]. However, the question of what determines the factors contributing to this relationship remains unanswered.

We hypothesized that athletes would present greater P300 amplitude and shorter reaction time when compared with non-athletes. The state of the art demonstrated that no study has examined the combinatorial relationship of P300 and saccadic go/no-go task for comparisons between volleyball players’ and non-athletes. In this respect, volleyball was chosen because successful performance in this sport requires a wide range of perceptual-motor skills, e.g., visual discrimination abilities, visual search skills, central-peripheral awareness, visual concentration and especially short reaction times in responding to environmental demands [3]. Thus, this preliminary study contributes to knowledge about neural mechanisms underlying attentional processing during sports performance, and may influence the construction of intervention strategies to sports performance and reassert the use of sport as a resource to improve attention performance in non-athletes.

## 2. Material and methods

### 2.1. Participants

We recruited thirty-one healthy volunteers (4 men, 11 women), with age from 12 to 17 years (mean ± standard deviation [SD] = 16.9 ± 0.3 years) in this preliminary study. The subjects were divided into two groups: sixteen volleyball athletes (15.8 ± 0.2 years) and fifteen non-athletes as a control group (16.2 ± 0.3 years). An Independent *t*-test was performed between the two groups for age and showed no significant difference (p>0.05). Only right-handed individuals were selected based on the Edinburgh Inventory [28] and had normal or corrected-to-normal vision. None reported a history of psychiatric or neurological disorders nor a history of use of psychoactive or psychotropic substances.

To estimate the sample size, we searched for previous studies with an experimental sample design similar to ours [7,10]. We analyzed the average number of participants, and we decided to apply a larger sample number than that of previous studies. The athletes were recruited from a professional volleyball club, and healthy controls from schools close to the university campus were recruited for the control group.

We analyzed the Sustained Attention Test (SA) to investigate if the functions of concentrated attention, speed with quality, and support of attention were comparable [29]. The Sustained Attention test was applied to ensure that all participants did not present any impairment in the functioning of attention; this test was used as a selection criterion for the study. An Independent *t*-test was performed between the two groups for the SA test and showed no significant difference (p>0.05), with Cronbach’s alpha (α) = 0.79; p = 0.001. The results demonstrated no differences for neuropsychological tests of attention (concentration p = 0.70 ± 6.5, total hits p = 0.40 ± 4.0; speed with quality p = 0.40 ± 8.1).

SA test description: The tests were corrected using the total number of correct answers, errors, and omissions. During the test, participants were submitted to a sequence of visual stimuli (target and non-target figures) and asked to mark the target stimuli with a risk. The test is performed with a pencil and an answer sheet and consists of 25 rows with 25 stimuli each. The subject must select only one type of stimulus among the possibilities. The participant has 15 seconds to complete each row. At the end of the established time, the applicator gives the command to go to the next one, and, in this way, the participant immediately goes to the following line starts again. On average, the application time is 10 minutes.

All participants had normal vision, in addition, were not using any substance that could influence brain activities (e.g. tobacco, coffee, alcoholic beverages, caffeine-containing foods, or medications) 14h before or during the study period. Routine ophthalmologic examinations confirmed that all participants had normal visual function. The participants underwent a medical evaluation to exclude neurological or motor diseases and contraindications to the experimental procedure. The athletes in the study were recruited from the Brazilian national volleyball team and practiced volleyball for around 5.0 ± 2.8 years. The non-athletes not involved in any regular, sports activity.

Ethics approval was obtained from the local ethics committee. The participants signed a Free and Informed Consent Declaration under the ethical standards established in the Helsinki Declaration, 1964.

### 2.2. Experimental procedure and task

**Fig 1:**
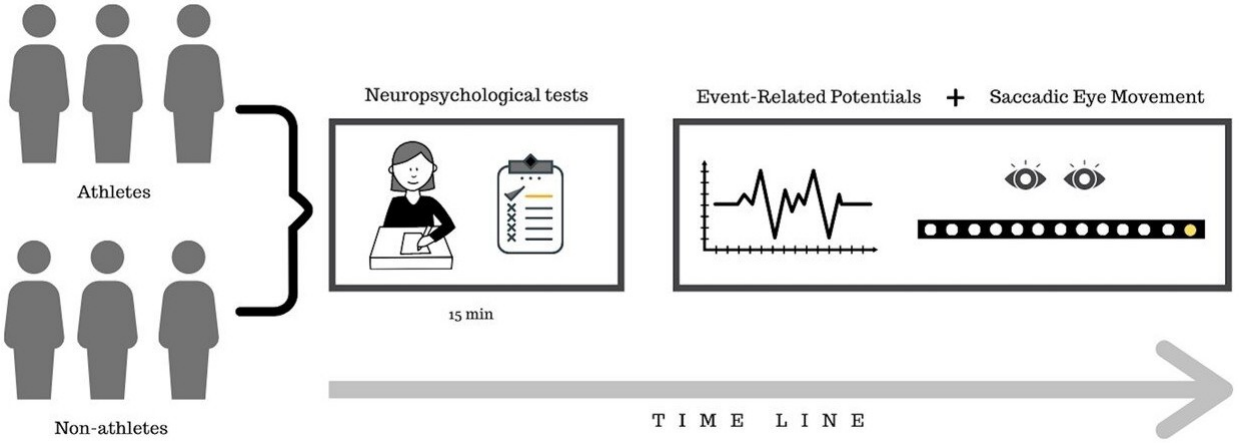
Experimental design.

The participants were accommodated in a room with brightness control, sound isolation, and electrical grounding. A 120 cm length bar composed of 13 light-emitting diodes (LEDs) was placed 100 cm away from the participant’s eye level (Fig 1). The bar had a central warning (bi-color LED–green and red) and six more LEDs located on each side (6 LEDs located on the left side of fixation and 6 LEDs located on the right side). The distance between the participants’ eyes and the LED bar was standardized at 100 cm. The computer software–SEM Acquisition controls the LED bar determining the presentation of the stimulus and measure the reaction time to perceive the visual stimulus. The reaction time recording is associated in combination with the ocular electrical activity, or electrooculogram (EOG), which captured by the placement of two 9 mm diameter electrodes mounted bipolarly. The electrodes were placed in the outer corner of the left and right eyes that recorded the horizontal eye movements (hEOG).

**Fig 2:**
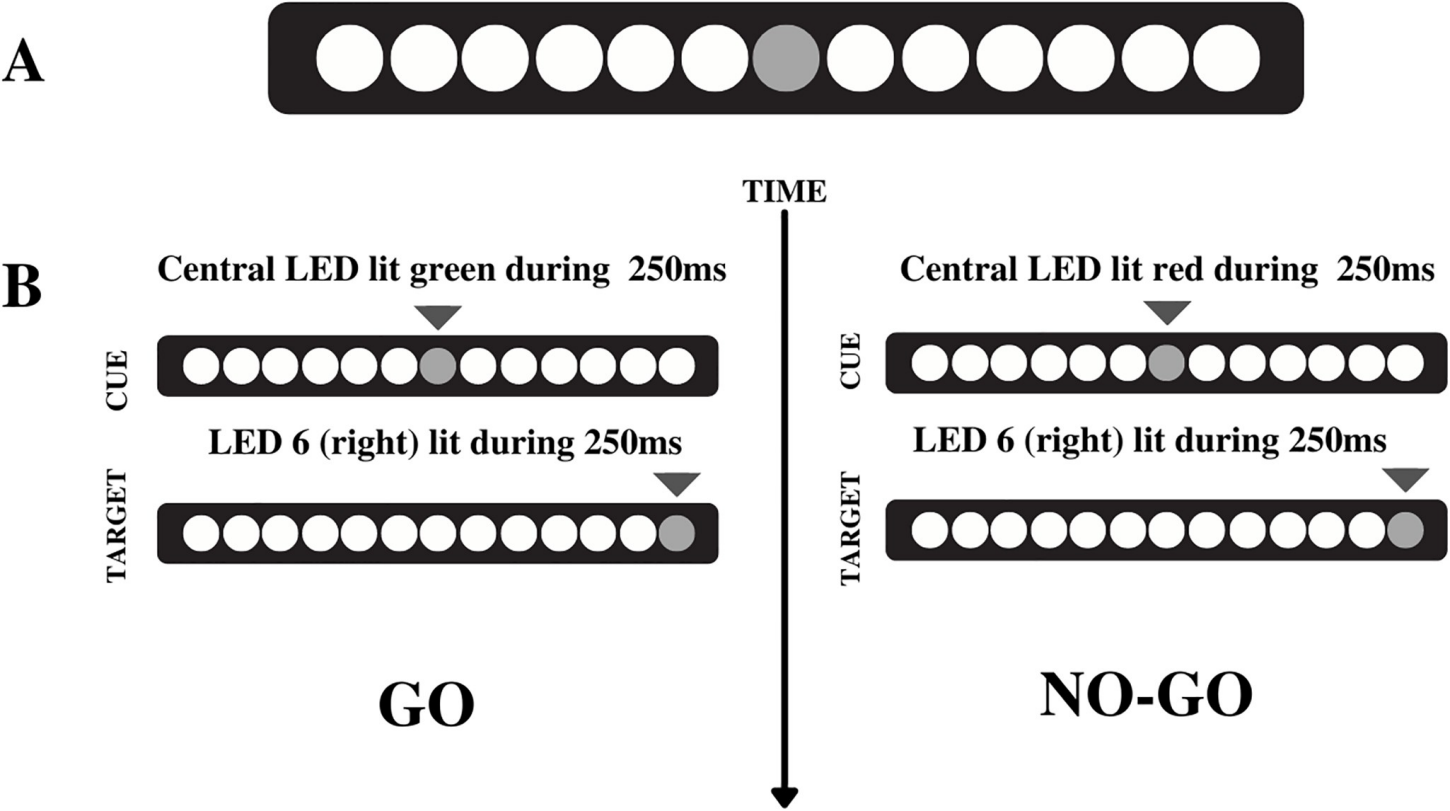
LED stimuli.

We analyze the EEG acquisition during the task execution. All subjects performed one block with 120 trials at their own pace during of the SEM paradigm. The procedure consisted with ten target-LED lights placed on the right side of the bar and 10 LED lights on placed the left side of the bar from the center. During the experimental procedure, the participants were instructed to keep their eyes fixed on the center of the bar and shift their eyes when they perceived one of the diodes lighting up, no head movements, only eye movements. The saccadic eye movement (SEM) paradigm consisted of a fixed pattern of stimulus presentation where the target-stimulus (target-LED) always appeared randomly between left and right sides at a predefined position, this condition was characterized by the predictability of the appearance of the stimulus in time and space, being considered directed by memory. The paradigm is characterized by predictability since the stimulus appears at a predefined spatial location in the periphery of the visual field. Each LED remained lit for 250 ms, with an inter-LED time of 2 seconds (Fig 2).

### 2.3. EEG recording

All subjects were accommodated in a room with acoustic insulation, electrical grounding, and low light. Subjects sat in a chair with armrests to minimize muscle artifacts during EEG signal acquisition. The 20-channel continuous EEG was recorded by BrainNet BNT36 (EMSA Medical Equipment). The silver/silver chloride electrodes were positioned through a nylon cap following the international 10–20 system, including binaural reference electrodes (SPES Medical Brazil). The EEG electrodes impedance and EOG electrodes were kept below 5kΩ. The acquired data had an amplitude below 100 μV. The sampling rate was 240 Hz. An antialiasing low-pass filter with a cut-off frequency of 100 Hz was employed. It was configured to use 60 Hz Notch digital filtering, with highpass filters at 0.03 Hz and low pass filters at 40 Hz (Order 2 Butterworth filter), using the Data Acquisition software (Delphi 5.0).

The signal corresponding to each EEG derivation came from the electric potential difference between each electrode and the pre-set reference (earlobes). The epochs were time-locked to the stimulus presentation, and we extracted 15 epochs for each participant before the stimuli appearance and 15 more epochs after the stimuli presentation.

The electrodes located on the central line of the head, Fz, Cz and Pz were selected due to their relationship with the interconnectivity pattern of left and right hemispheres better related to neurobiological process [30,31].

### 2.4. EEG data processing

A visual inspection and independent component analysis (ICA) was applied to identify and remove all remaining artifacts through Matlab 5.3^®^ (The Mathworks, Inc.). Data from individual electrodes that showed contact loss with scalp or high impedance (>5kΩ) were not considered. After ICA, the overall rate of removal for noisy data in each participant was less than ten percentage independent of the task condition. A classical estimator (i.e., parametric, Bartlett Periodogram, using non-overlapping 2 s long [480 samples] rectangular windows) was applied to the Power Spectral Density (PSD), estimated from the Fourier Transform (FT), which was performed using MATLAB (Mathworks, Inc.). Epochs were selected between 1-sec pre-stimulus to 1.5-sec post-stimulus. The total number of epochs used after visual inspection and ICA for each group was as follows: non-athlete group (n = 376 epochs); athlete group (n = 366 epochs).

After specific channels were selected (Fz, Cz, and Pz), the event-related potentials (ERPs) transform was computed for the electrodes. The data were averaged and represented graphically in terms of latency (x-axis) and amplitude (y-axis). In the context, the P300 component was identified as the most positive component within the latency window of 250–500 ms. Amplitude was measured relative to a pre-stimulus baseline, with peak latency defined as the time point of maximum positive amplitude within the specific latency window.

### 2.5. Statistical analysis

Statistical procedures were conducted using IBM SPSS for Windows (version 21.0; IBM, Armonk, NY, USA). Analyses were controlled for age using an Independent *t*-test, where no significant effects were found. The normality and Homogeneity of variance of the data were previously verified by the Shapiro–Wilk and Levene tests. We use the data as mean, standard deviation (SD), and standard error (SE). The differences in the P300 amplitude and the reaction time for the SEM task were analyzed by Independent t-tests between athlete and non-athlete groups, with the analysis effect evaluated by Cohen’s d. The effect sizes were calculated (≤0.039: no effect, 0.04–0.24: minimum, 0.25–0.63: moderate, ≥0.64: strong, according to Ferguson (2009). For all statistical analyses, the significance level was α = 0.05 [32,33].

## 3. Results

### 3.1. Reaction time

The analysis by Independent *t*-test showed statistical difference [*t*(1) = 4.71; p = 0.001; d = 0.21; CI95% = 1.14–2.91], with a lower reaction time in the athlete group (mean: 317.84ms, SD: 58.68ms, SE: 1.92ms) when compared to non-athlete (mean: 330.99ms, SD: 52.49ms, SE: 1.83ms) (Table 1). In addition, the findings revealed a minimum effect for behavioral measure.

**Table 1:** Reaction time for athlete (left) and non-athlete (right) for the saccadic eye movement (SEM) paradigm. Differences of mean significance in reaction time were obtained by mean and standard deviation, and the statistically significant differences (p = 0.001) are indicated with (*).

	Athlete group		Control group		
Data	Mean	± SD	Mean	± SD	p-value
Reaction time (ms)	325	58	330	52	p = 0.001*

### 3.2. P300 event-related potentials

**Fig 3:**
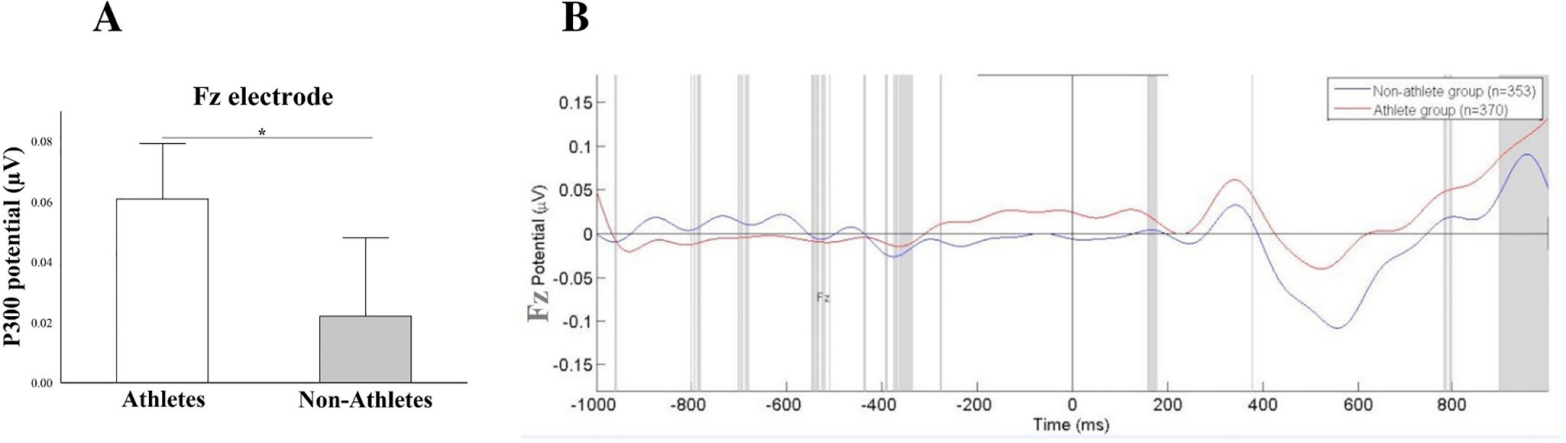
A) Difference for P300 under Fz electrode. The results are represented by the mean ± standard error, and the statistically significant differences (p = 0.021) are indicated with (*). The athletes increase the P300 potential compared with non-athletes; B) Mean Potential (μV) of visual P300 ERPs from the athlete’s group (red line) and non-athletes (blue line) in response to the Leeds stimulus obtained from the Fz electrode.

**Fig 4:**
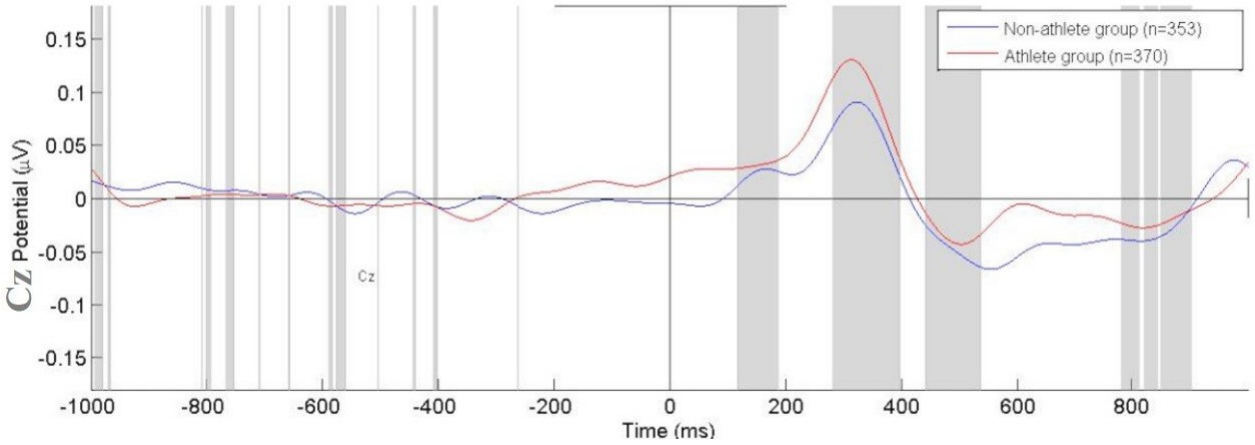
Mean Potential (μV) of visual P300 ERPs from the athletes group (red line) and non-athletes (blue line) in response to the Leeds stimulus obtained from the Cz electrode.

**Fig 5:**
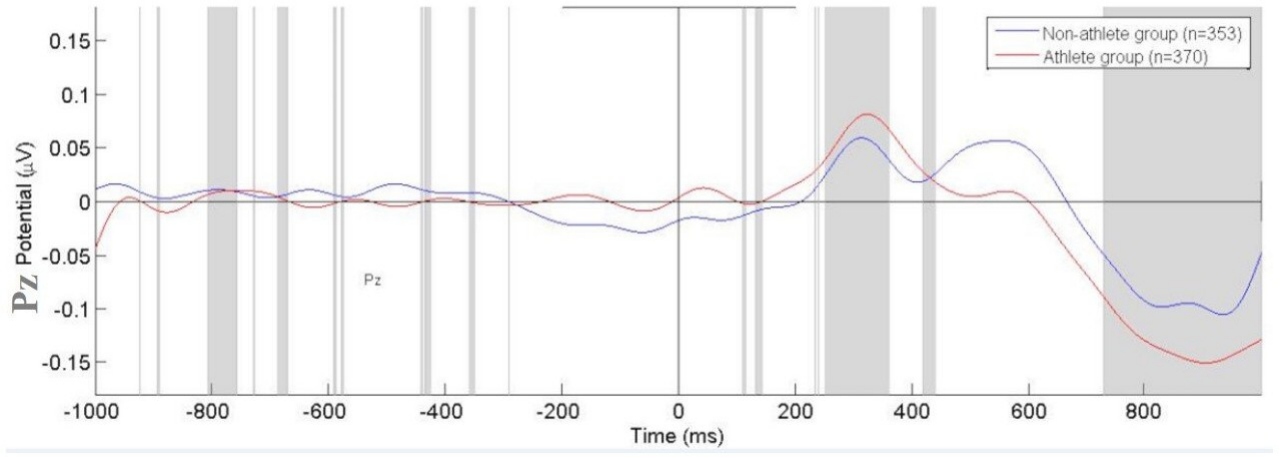
Mean Potential (μV) of visual P300 ERPs from the athletes group (red line) and non-athletes (blue line) in response to the Leeds stimulus obtained from the Pz electrode.

The analysis of P300 event-related potentials by Independent t-tests for the Fz electrode revealed the difference between groups, with [*t*(1) = 4.43; p = 0.021; d = 0.23; CI95% -0.008–0.086] (Fig 3). The athletes group had a greater mean potential of P300 ERPs when compared to the non-athletes. In relation to Cz and Pz electrodes, no statistical difference was found (p>0.05). We demonstrated the target ERPs amplitude of each electrode inspected (Fz, Cz, and Pz). The mean potential (μV) of visual P300 ERPs from the athlete’s group and non-athletes was demonstrated in response to the visual stimulus (13 light-emitting diodes) for the SEM task (Figs 4, 5 and Table 2).

**Table 2:** Difference for P300 under Fz, F3, F4, Cz e Pz electrodes. The results are represented by the mean ± SD, and the statistically significant differences are indicated with (*).

	Athlete group		Control group		
Amplitude (μV)	Mean	± SD	Mean	± SD	p value
Fz	0.06	0.02	0.03	0.01	0.185
F3	0.22	0.17	0.17	0.14	0.006*
F4	0.18	0.14	0.30	0.22	0.000*
Cz	0.12	0.08	0.09	0.05	0.151
PZ	0.08	0.04	0.05	0.02	0.183
Latency (ms)	Mean	± SD	Mean	± SD	p value
Fz	338	40	340	58	0.34
F3	353	53	318	44	0.13
F4	358	69	366	62	0.21
Cz	311	55	321	61	0.16
Pz	323	40	313	46	0.12

## 4. Discussion

We analyzed the differences in the electrocortical activity by P300 ERP component and reaction time, comparing volleyball athletes and non-athletes. The hypothesis for differences in P300 was partially confirmed in this preliminary study. In relation to reaction time, the findings showed a minimum effect, with a shorter reaction time for athletes.

The findings for reaction time corroborate with highly trained skills in high-level sports. We can consider that volleyball athletes need to process information from both central and peripheral vision, and the recurrence of training can adjust the visual focus, improving reaction time during binocular, dominant eye, and no dominant eye viewing conditions [26,34]. Since athletes respond faster to visual stimuli presented during a go/no go sensorimotor integration task. The recurrence of task training improves neural synchronization during the adjustment motor to respond to stimuli, whether visual, auditory, or nociceptive.

The reaction in the decision-making triggered by visual stimuli is a part of dynamic interactions between personal experience and environmental conditions and has been widely correlated with motor strategy [34]. Soon, the reaction time through saccadic eye movement paradigm tasks involves a complex mechanism of inputs and outputs in cortical regions during the scales timing at the sub and supra-second levels, involved in feed-forward systems for which sensory input is used to improve movement accuracy [35,36].

Since reaction time functions involve recurrent interactions with external surroundings, the behavioral tasks can plausibly improve muscle response for a given objective. In this context, Giglia et al. [37] showed shorter reaction time in the open ability players group, which included volleyball athletes, than in the closed and sedentary skill group. This may be possible via the dopaminergic modulation in the connections of the basal ganglia with the cortical areas interconnectivity pattern of left and right hemispheres, which are related to the sensory-motor integration of the neurobiological process of attention [38]. Our inferences are supported on the ability of the saccadic eye movement tasks to encode changes in neuronal connections distributed in neural networks associated with adjustment and motor control (frontal, parietal and primary motor area). In addition, studies with badminton players have also shown a main effect of group, with the reaction time during a flanker task being lower for athletes than controls [38,39]. The differences for reaction result from neural inhibition for non-task-matched stimuli; this increases attentional recruitment and generates faster response stimuli [35].

Previous studies show the effect of sports training in electroencephalographic results [3,4,38,39]. The hypothesis of differences in P300 ERPs between athletes and non-athletes during visual processing, states modulations in cortical energy consumption, since expert athletes can achieve better performance with less neural activity than non-athletes, which means neural efficiency [4].

The visual modulation might be the result of the specific requirements for a given sport training. Our findings demonstrated differences during the sensory processing only for the frontal midline, which may corroborate the precepts that athletes have more efficient neural response rates in the perception of visual stimuli [40,41]. The increase of the P300 amplitude in athletes than non-athletes suggest adjustment for the neurobiological functions of the attentional level for the stimulus, as well as the decision-making of response to the same [25,40,42].

This difference can evidence faster neural signal transmission in the visual focus [23]. The greater P300 potential during the saccadic eye movement paradigm tasks seems to occur due to lower cognitive demand to initiate the task, which is needed to create an internal model for planning, speed and execution [43]. This can be associated with working memory and attentional level [44], both related to the frontal area, which works as an integrating center of the neural inputs during visual perception task.

The visual architecture needs to provide information predicting when the stimuli will arrive [45]. In focus the volleyball, for effective response selection and action execution players must process and integrate a large amount of dynamic visual information, including flight information of the ball and kinetic information of the opponent. Thus, the P300 modulation in the frontal area might be the result of the specific requirements for a given sport training. In particular, the modulation of early sensory processing seems to be evident in athletes involved in ball sports requiring rapid responses to visual stimuli [3,21].

However, our findings no demonstrated differences and effects for motor and parietal interconnection area during rapid responses to visual stimuli. The findings of no difference in P300 activity can be evidenced by the difficulty of discriminating a target in a visual paradigm, which could affect the scalp topography [18]. When target stimuli occur in a series of more non-targets, no significant P300 component is elicited over the parietal or central scalp areas [18,46].

The variability between genders, as well as heterogeneity within and between groups, were limitations of this preliminary study. In addition, the sample size is a limiting factor, interfering with our results. Previous studies showed a low statistical power because the low signal-to-noise rates and the small sampling impact the results [19,47]. Another limitation was using 20-channel EEG (BrainNet BNT36 EMSA Medical Equipment). Despite the lower spatial resolution, using fewer channels is essential to avoid communication between electrodes, a variable that must be considered when seeking accurate recordings of the electrophysiological signal [48,49]. However, the investigation of reaction time in volleyball athletes resulted in significant findings that may contribute to the knowledge of specific cognitive functions of this sport. It may encourage the development of different training strategies to increase performance.

## 5. Conclusion

Our findings suggest that volleyball athletes demonstrate an allocation of attention to process the visual stimulus during the saccadic response task and a shorter reaction time in the responses when compared to non-athletes. Therefore, this approach correlates the hypothesis of neural efficiency and the respective effects of sports training on the behavioral activity of athletes.
